# Carvacrol protects mice against LPS-induced sepsis and attenuates inflammatory response in macrophages by modulating the ERK1/2 pathway

**DOI:** 10.1038/s41598-023-39665-7

**Published:** 2023-08-07

**Authors:** Chenghua Yan, Wendong Kuang, Liang Jin, Rongliang Wang, Ling Niu, Chuanqi Xie, Jian Ding, Yongcui Liao, Liyuan Wang, Hongjiao Wan, Guangqiang Ma

**Affiliations:** 1 College of Traditional Chinese Medicine/College of Life Sciences, Jiangxi University of Chinese Medicine, Nanchang, 330004 China; 2 Institute of Microbiology, Jiangxi Academy of Sciences, Nanchang, 330029 China; 3 State Key Laboratory of Virology, Wuhan, 430071 China; 4 Institute of Applied Chemistry, Jiangxi Academy of Sciences, Nanchang, 330029 China; 5 The First Affiliated Hospital of Nanchang University, Nanchang, 330006 China

**Keywords:** Immunology, Inflammation, Sepsis

## Abstract

Macrophages play an important role in the development of life-threatening sepsis, which is characterized by multiorgan dysfunction, through their ability to produce inflammatory cytokines. Carvacrol is a phenolic compound that has been confirmed to possess strong anti‑inflammatory activity. In this study, we mainly investigated the effect of carvacrol on lipopolysaccharide (LPS)-induced macrophage proinflammatory responses and endotoxic shock. The results showed that carvacrol significantly reduced mouse body weight loss and ameliorated pathological damage to the liver, lung, and heart under LPS-induced sepsis. Carvacrol attenuated inflammatory responses by inhibiting the LPS-induced production of inflammatory cytokine interleukin-6 (IL-6) in vivo and in vitro. Mechanistically, carvacrol inhibited IL-6 production mainly through the ERK1/2 signalling pathway in macrophages. Furthermore, carvacrol improved the survival of septic mice. This study sheds light on the role of carvacrol in the pathogenesis of LPS-induced sepsis, and thus, its potential in treating sepsis patients may be considered.

## Introduction

Sepsis, defined as “life-threatening organ dysfunction caused by a dysregulated host response to infection,” is characterized by an excessive inflammatory response associated with high mortality^1–3^. Sepsis can occur due to microbial products, such as pathogen-associated molecular patterns (PAMPs) and damage-associated molecular patterns (DAMPs), which cause multiple organ injuries^4^. As a well-known heterogeneous disease, the underlying molecular mechanism of sepsis, especially the host response and pathogenesis during the early stage of the disease, has been intensively researched. Many studies have focused on predictive and prognostic markers that can be used during the early stages of sepsis to determine effective treatment^5, 6^. The latest research revealed that coagulation factor protein levels in the preseptic state predicts the dysregulation of fibrinolytic and anti-coagulant activities that arise during murine sepsis^7^. Moreover, one study demonstrated that intestinal fungi play a protective role in the pathogenesis of sepsis, and the use of antifungal drugs can promote the occurrence of sepsis^8^. Another study found that 6-gingerol improved sepsis-induced immune dysfunction by regulating cytokine balance and reducing lymphocyte apoptosis^9^. A better understanding of sepsis pathology and the development of new therapies are essential to meaningfully improve the status of sepsis care.

Carvacrol is a natural monoterpene phenol found in the essential oils of thyme and oregano and is derived from the *Origanum* and *Thymus* genera or other medicinal plants^10^. Carvacrol exhibits various pharmacological activities, including antimicrobial^11^, antitumour^12^, and anti-inflammatory^13^ effects. Due to its important antibacterial and anti-inflammatory functions, it has a strong curative effect on a variety of diseases, especially inflammatory diseases, and has thus become a research hotspot. One study reported that carvacrol ameliorates acute campylobacteriosis by reducing the serum levels of interferon-gamma (IFN-γ), TNF, monocyte chemoattractant protein-1 (MCP-1), and IL-6^14^. In addition, carvacrol significantly reduced the expression of immunoglobulin E (IgE), TNF-α and IL-4, while it significantly increased the expression of superoxide dismutase (SOD) and glutathione (GSH) in ovalbumin-induced inflammatory disease asthma^15^. Carvacrol prevents elevations in the levels of the inflammatory cytokines TNF-α and IL-6 and protects organs from damage in a murine model of caecal ligation and puncture-induced polymicrobial sepsis^16^. Besides, Carvacrol inhibits the production of proinflammatory cytokines such as IL-6 and IL-17 and increases the production of anti-inflammatory cytokines such as transforming growth factor-β (TGF-β) and IL-10 in EAE and exhibits anti-inflammatory properties^17^. Moreover, several studies have reported that carvacrol has protective effects against acute lung injury, inhibiting the production of inflammatory cytokines in serum and reducing oxidative stress in LPS-induced sepsis^18^. However, the effects of carvacrol on multiple organ damage and inhibition of macrophage activation remain unclear. Therefore, we first studied the role of carvacrol in LPS-induced multiple organ damage and macrophage activation and then explored its possible mechanism.

In this study, we observed that carvacrol reduced the mortality of septic mice, inhibited the LPS-induced production of proinflammatory cytokine IL-6 in serum, and ameliorated the pathological injury of liver, lung and heart. In addition, carvacrol significantly inhibited the production of the inflammatory cytokine Il-6 through the extracellular regulated protein kinase (ERK1/2) signalling pathway in macrophages.

## Results

### Carvacrol reduced LPS-induced body weight loss

**Figure 1:**
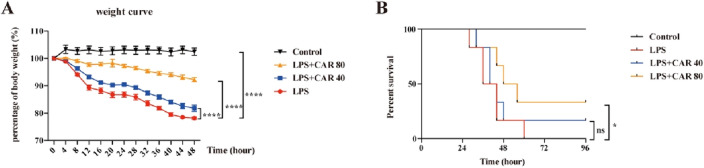
Carvacrol protected mice from endotoxic shock. We treated C57BL/6 mice with carvacrol (40, 80 mg/kg of body weight) or 0.2% Tween 80, followed by LPS stimulation. (**A**) Mouse body weight was measured (n = 7 mice/group) after LPS (10 mg/kg of body weight) stimulation. (**B**) Observed mouse survival (n = 10 mice/group) after LPS (25 mg/kg of body weight) stimulation. Data were analysed by two-way ANOVA with Bonferroni post-test. The error bars show the means ± SEMs. *P < 0.05; ****P < 0.0001 and *ns* not significant.

In this study, we focused on elucidating the role of carvacrol in the proinflammatory response of macrophages and the pathogenesis of sepsis. To investigate the role of carvacrol during inflammation, we subjected the mice to sublethal doses of LPS. See the supplementary material for details regarding the groupings (Fig. S1). First, we investigated the body weight loss of mice treated with different doses of carvacrol under LPS-induced endotoxic shock. In accordance with our prediction, carvacrol reduced the body weight loss of the mice induced by LPS (Fig. 1A). To investigate whether carvacrol affects the survival of LPS-treated septic mice, mice were intraperitoneally administered different doses of carvacrol for 2 h before LPS injection, and the death of the mice was observed. As shown in Fig. 1B, LPS-treated mice given carvacrol had a significantly longer survival time and a better survival rate than LPS-treated mice given 0.2% Tween 80. The data revealed that carvacrol reduced the mortality of LPS-treated mice, further demonstrating that carvacrol protected the mice from endotoxic shock.

### Carvacrol alleviated tissue injury under LPS-induced sepsis

**Figure 2:**
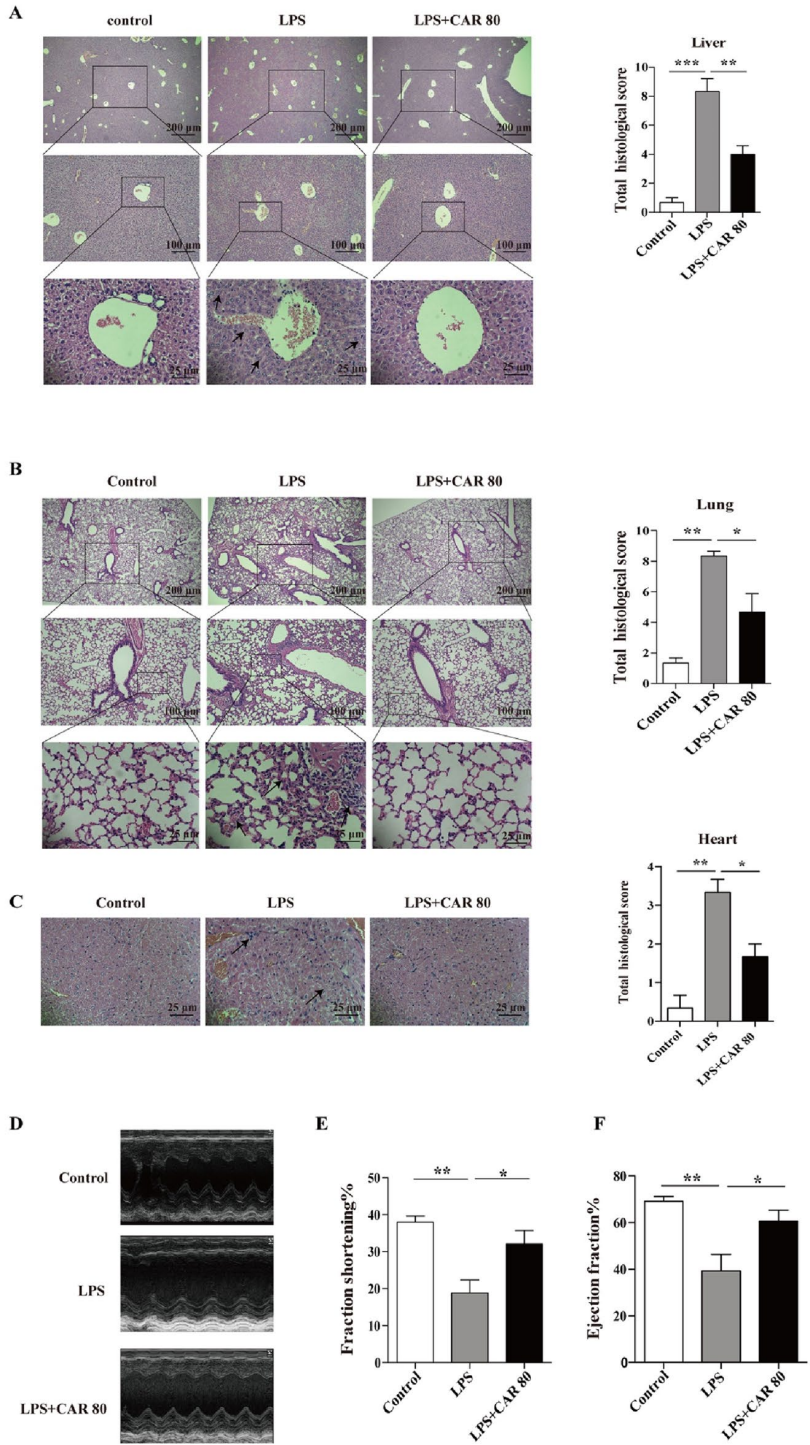
Carvacrol alleviated mouse liver, lung and heart injury in LPS-induced sepsis. (**A**,**B**) We treated C57BL/6 mice with carvacrol (80 mg/kg of body weight) or 0.2% Tween 80 for 2 h and then used LPS (10 mg/kg of body weight) for stimulation. Lung, liver, and heart samples were harvested and fixed with 4% paraformaldehyde at 12 h after LPS injection. Staining of paraffin-embedded liver (**A**), lung (**B**), and heart (**C**) sections with haematoxylin–eosin staining (H&E) and histological analysis of acute liver injury (the arrow marks cytoplasmic colour fading, vacuolization, nuclear condensation, nuclear fading), lung injury (the arrow marks haemorrhage, lung oedema, inflammatory cell infiltration) and heart injury (the arrow marks myocardial cell structure and inflammatory cell infiltration) (n = 3 mice/group). (**D**) Representative echocardiographic images from each group. (**E**,**F**) The ejection fraction and fraction shortening of the heart were measured using the images in (**D**). Data were analysed by one-way ANOVA with Tukey’s multiple comparisons test. The error bars show the means ± SEMs. *P < 0.05; **P < 0.01; ***P < 0.001.

LPS-induced endotoxic shock is a systemic inflammatory response syndrome, and excessive inflammation can lead to tissue damage^19, 20^. To evaluate whether carvacrol influenced this process, we evaluated tissue damage in the carvacrol-treated mice subjected to LPS stimulation. The mice were pretreated with carvacrol for 2 h and stimulated with LPS for 12 h. Lung, liver, and heart samples were collected, and the pathological lesions were observed by HE staining. We observed much less damage in the liver, lung, and heart of carvacrol-treated mice (Fig. 2A–C); the levels of inflammatory cell infiltration, haemorrhage, and oedema were reduced in the lung tissue; the cytoplasmic colour fading, vacuolization, nuclear condensation, and nuclear fading of the liver cells were significantly reduced; and the levels of myocardial cell structural changes and inflammatory cell infiltration were also reduced in the heart. To further investigate whether carvacrol improved cardiac function in mice subjected to endotoxic shock, we assessed the ejection fraction (EF) and fractional shortening (FS) by echocardiography (Fig. 2D–F). The data showed that LPS-treated mice exhibited significantly lower EF and FS than mice in the control group, and those subjected to pretreatment with carvacrol had higher EF and FS than those in the LPS group. These results imply that carvacrol provides an indispensable function of organ protection under exposure to LPS.

### Carvacrol inhibited the proinflammatory response of macrophages in septic mice

**Figure 3:**
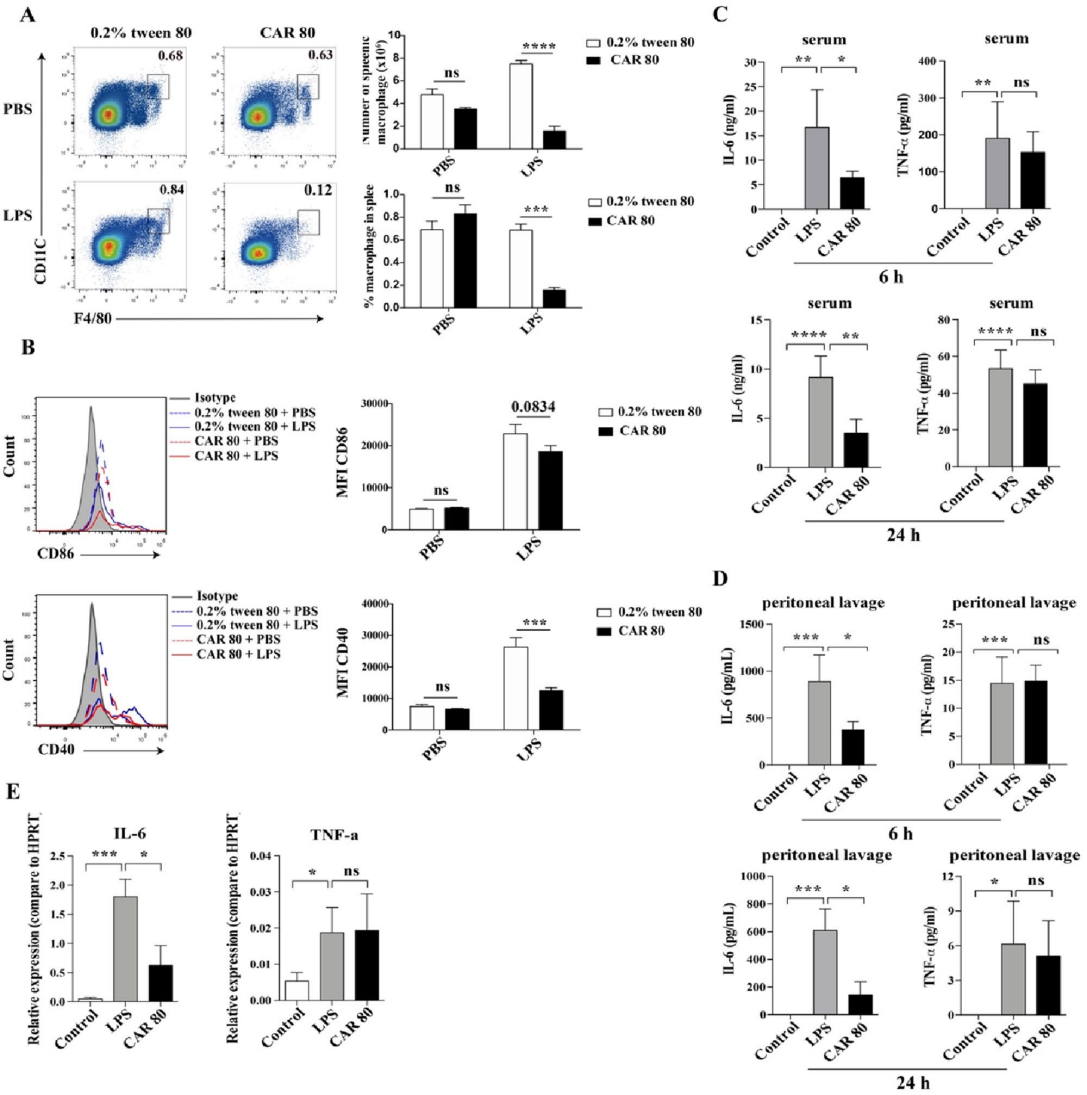
Carvacrol inhibited LPS-induced macrophage activation and the proinflammatory response in vivo. We treated C57BL/6 mice with either carvacrol (80 mg/kg of body weight) or 0.2% Tween 80 for 2 h, followed by LPS (10 mg/kg of body weight) stimulation. (**A**) Flow cytometry analysis of cell numbers and percentages of CD11b^+^F4/80^+^ macrophages in the spleen after stimulation with LPS or PBS for 12 h (n = 4 mice/group). (**B**) Representative flow cytometry analysis of the levels of CD86 and CD40 and the mean fluorescence intensity of CD86 and CD40 in spleen macrophages as in (**A**). ELISA analysis of IL-6 and TNF-α in the serum (**C**) or peritoneal lavage (**D**) from carvacrol- or 0.2% Tween 80-treated mice 6 or 24 h after LPS exposure (n ≥ 3 mice/group). (**E**) IL-6 and TNF-α mRNA expression in mouse peritoneal cells was measured by RT‒qPCR (n = 3 mice/group). Data were analysed by two-way ANOVA with Bonferroni posttest (**A**,**B**) or one-way ANOVA with Tukey’s multiple comparisons test (**C**–**E**). The error bars show the means ± SEMs. *P < 0.05; **P < 0.01; ***P < 0.001; ****P < 0.0001 and *ns* not significant.

To explore whether the protective effect of carvacrol against sepsis occurred by reducing the number of LPS-exposed macrophages, we first measured the percentage and number of splenic macrophages. The results showed that mice administered carvacrol exhibited a significantly reduced percentage and number of splenic macrophages following LPS exposure (Fig. 3A). To evaluate whether carvacrol inhibited the activation of macrophages, we examined CD86 and CD40 expression. The expression of CD40 in the macrophages of mice given carvacrol was significantly lower than that in mice given 0.2% Tween 80. In contrast, CD86 expression displayed no significant difference under the same conditions (Fig. 3B). To further elucidate the role of carvacrol in macrophage function, we next examined the levels of proinflammatory cytokines in mouse serum and peritoneal cells. It was interesting to observe that with carvacrol administration, the levels of serum IL-6 but not TNF-α were significantly reduced after LPS injection for 6 h or 24 h (Fig. 3C). Notably, the same effect could be achieved by excessive peritoneal lavage (Fig. 3D). RT-qPCR analysis showed that carvacrol treatment significantly reduced IL-6 mRNA levels in LPS-stimulated mouse peritoneal cells (Fig. 3E). Neither the carvacrol- nor 0.2% Tween 80-treated groups displayed any significant difference in terms of the protein level and mRNA level of TNF-α. Generally, these observations suggest that carvacrol negatively regulates proinflammatory responses in macrophages under LPS-induced endotoxic shock.

### Carvacrol inhibited the proinflammatory response of LPS-stimulated macrophages in vitro

**Figure 4:**
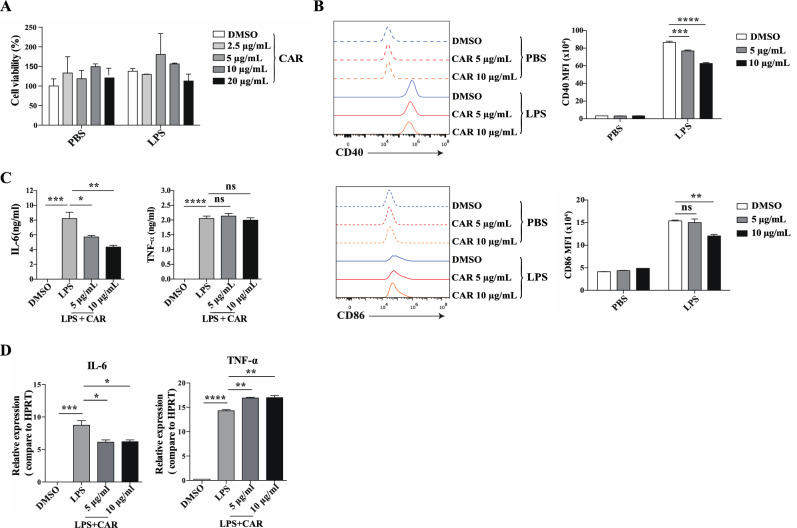
Carvacrol inhibited the LPS-induced macrophage proinflammatory response in vitro. (**A**) BMDMs were pretreated with different concentrations of carvacrol (0–20 μg/mL) and then treated with PBS or LPS for 24 h, and cell viability was assessed by CCK8 assay. (**B**–**D**) BMDMs were pretreated with different doses of carvacrol (0, 5, and 10 μg/mL) for 2 h and then treated with LPS (1 μg/mL). (**B**) FACS analysis of CD86 and CD40 expression in BMDMs after stimulation with LPS for 24 h. (**C**) After LPS stimulation for 24 h, the protein levels of IL-6 and TNF-α in the supernatant were measured by ELISA. (**D**) After 6 h of LPS stimulation, the mRNA levels of IL-6 and TNF-α were measured by RT‒qPCR. Data were analysed by two-way ANOVA with Bonferroni posttest (**A**,**B**) or one-way ANOVA with Tukey’s multiple comparisons test (**C**,**D**). The error bars show the means ± SEMs. *P < 0.05; **P < 0.01; ***P < 0.001; ****P < 0.0001 and ns: not significant.

To further explore the protective effect of carvacrol on the LPS-induced macrophage proinflammatory response, we used BMDMs to evaluate the effect of carvacrol in vitro. Almost 100% of BMDMs differentiated from bone marrow were CD11b^+^F4/80^+^ positive cells (Figure S2). First, we assessed the viability of cells that had been treated with different doses of carvacrol for 24 h. As the concentration of carvacrol increased, the activity of cells was not affected (Fig. 4A), indicating that carvacrol within 20 µg/mL does not affect cell viability. Next, we selected 5 µg/mL and 10 µg/mL carvacrol to treat BMDMs for 2 h and then simulated them with LPS to examine the effect of carvacrol on CD86 and CD40 expression. After LPS stimulation, the cells treated with carvacrol exhibited significantly lower expression of CD40 than dimethyl sulfoxide (DMSO)-treated cells. At a carvacrol concentration of 10 µg/ml, CD86 expression changed significantly (Fig. 4B). Additionally, incubating BMDMs with carvacrol resulted in decreased expression of IL-6 at both the protein and mRNA levels. In addition, the mRNA but not the protein levels of TNF-α increased (Fig. 4C,D). In summary, all these data show that carvacrol negatively regulates LPS-induced proinflammatory responses primarily in macrophages.

### Carvacrol inhibits the ERK1/2 signalling pathway activated by LPS

**Figure 5:**
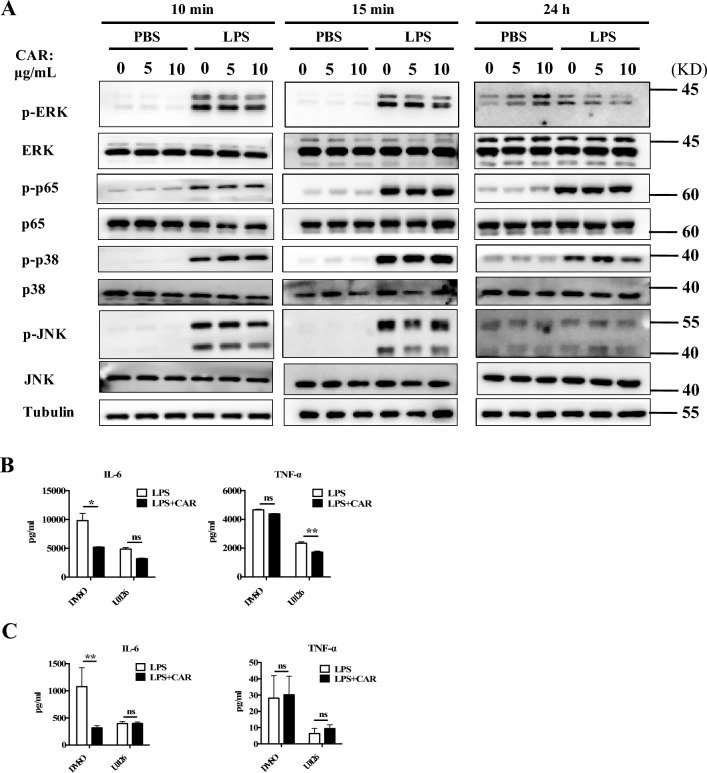
Carvacrol inhibits ERK1/2 signalling pathway activated by LPS. (**A**) We pretreated BMDMs with different doses of carvacrol (0, 5, and 10 μg/mL) for 2 h and then treated these cells with LPS (1 μg/mL) for 10 min,15 min or 24 h. Immunoblot analysis of whole-cell lysates was performed with the indicated antibodies. Tubulin was used as a control. (**B**) BMDMs were pretreated with U0126 (10 μM) for 1 h, followed by carvacrol (10 μg/mL) for 2 h, and then administered LPS (1 μg/mL) for 24 h. IL-6 and TNF-α levels were measured in the culture medium. (**C**) Mice were pretreated with carvacrol (80 mg/kg) for 2 h and then stimulated with LPS (5 mg/kg of body weight). After 1 h, U0126 (20 μL of 20 mM) was injected intraperitoneally (n = 4 mice/group). The grouping of gels/blots cropped from different parts of the same gel (p-p65, p-p38 and p-JNK; ERK and Tubulin; p65 and p38 for 10 min samples) or different gels (p-ERK and JNK for 10 min samples). For 15 min and 24 h samples, the grouping of gels/blots cropped from different parts of the same gel (ERK and p65; p-JNK and p-p38) or different gels (p-ERK, p-p65, JNK, P38 and Tubulin). The full blots are shown in Supplementary Information S1. Data were analysed by two-way ANOVA with Bonferroni post-test (**B**–**D**). *P < 0.05; **P < 0.01, and *ns* not significant.

To further explore the mechanism by which carvacrol inhibits LPS-induced inflammation, we pre-treated BMDMs with different doses of carvacrol and then exposed these BMDMs to LPS for 10 min, 15 min or 24 h. We observed that carvacrol only suppressed the phosphorylation of ERK1/2, but not that of p65, JNK, and p38, after LPS stimulation (Fig. 5A). We used specific inhibitor of MAPK (U0126) to pretreat BMDMs and evaluate the effect of carvacrol on LPS-induced inflammation. We found that U0126 inhibited the production of IL-6 and TNF-α in LPS-treated BMDMs. When cells were pretreated with U0126, the inhibitory effect of carvacrol on IL-6 production was significantly reduced (Fig. 5B). We also conducted in vivo experiments with the inhibitor and found that after treatment with U0126, the levels of IL-6 and TNF-α in the serum of septic mice were significantly reduced. When mice were treated with U0126, the inhibitory effect of carvacrol on LPS-induced IL-6 production was significantly reduced (Fig. 5 C). These data indicate that carvacrol mainly negatively regulates ERK1/2 signalling and subsequently inhibits the production of inflammatory factors and tissue damage after LPS stimulation.

## Discussion

Sepsis is a complex and rapidly developing inflammatory disease, and the molecular mechanism underlying the immune response to this condition warrants further exploration^21^. Sepsis results in many deaths every year and represents a major global health problem. Therefore, it is urgent to identify new therapeutic drugs for effective sepsis treatment. Macrophages play key roles in causing excessive inflammatory responses and organ failure and have important functions during all stages of sepsis^22, 23^. Therefore, modulation of macrophage immune function is of great importance for the treatment of sepsis^24, 25^. Many studies have revealed that natural products, such as astragalus polysaccharide^26^, mushroom polysaccharides^27^, and curcumin^28^ exert protective and therapeutic effects on sepsis. We identified that carvacrol attenuates the proinflammatory responses of macrophages and protects against septic shock in mice.

Carvacrol has been proven by numerous studies to exert anti-inflammatory activities^14, 17, 29^. Interestingly, we found that carvacrol prevents elevations in the levels of the inflammatory cytokines IL-6 not TNF-α in serum and peritoneal lavage and protects organs from damage in a murine model of LPS-induced sepsis. Besides, carvacrol inhibits the production of IL-6 not TNF-α in LPS stimulated bone marrow derived-macrophages in vitro. However, there are no reports on the effect of carvacrol on LPS-induced macrophage function. Whether carvacrol can attenuate LPS-induced inflammatory responses in macrophages in sepsis and its underlying mechanisms still need to be further explored.

Macrophages are one of the key types of cells involved in the pathogenesis of sepsis and exert effects by secreting inflammatory cytokines^30^. Carvacrol inhibits dendritic cell (DC) activation in vitro by decreasing the expression of CD40, which is also important for macrophage activation^31^. These studies indicate that carvacrol may protect mice from injury by inhibiting macrophage activation in the endotoxic shock model. We pre-treated cells with a certain concentration of carvacrol prior to LPS exposure and found that carvacrol effectively reduced the expression of CD40 and IL-6, which suggests that carvacrol can inhibit macrophage activation and proinflammatory cytokine production in vivo. The in vitro experimental results also showed that pre-treatment with carvacrol before LPS stimulation limited the activation and function of BMDMs.

Increasing evidence has shown that LPS-induced macrophage activation is mainly mediated by the ERK1/2 and NF-κB signalling pathways^32, 33^. LPS activates the phosphorylation of the mitogen-activated protein kinases ERK1/2, p38, JNK, and p65. Consistent with previous research showing that carvacrol suppressed LPS-induced RAW264.7 cell activation through the ERK1/2 and NF-κB pathways^34, 35^, we observed that carvacrol only significantly decreased p-ERK1/2 expression in BMDMs. Whether carvacrol interferes with LPS binding with TLR4 and the receptors for carvacrol warrants further study. In addition, both in vitro and in vivo results demonstrated that carvacrol reduced IL-6 expression but had no effect on TNF-α expression. Similar research found that javamide-II inhibits IL-6 production via suppressing the p38 signal pathway, without significant effects on the productions of TNF-alpha and IL-1beta in macrophage-like THP-1 cells, suggesting that carvacrol may regulate molecules that only affect IL-6 expression, and the specific molecular mechanism still needs further exploration.

In conclusion, the results of this study suggest that targeted modulation of the inflammatory cytokine storm with carvacrol could open new avenues for sepsis treatment strategies and demonstrate the potential application of carvacrol in pharmacological studies.

## Materials and methods

### Reagents and antibodies

LPS (#L2630, Escherichia coli 0111: B4) and carvacrol (#282197) were purchased from Sigma‒Aldrich (St. Louis, MO, United States). U0126 was purchased from MedChemExpress (#HY-12031). The following primary antibodies were purchased from Cell Signaling Technology: anti-p-NF-κB P65 (#3033), anti-p-ERK (#4370), anti-p-38 (#4092), and anti-p-JNK (#4668). M-CSF (#315-02) was obtained from PeproTech. The following reagents for flow cytometry were purchased from Thermo Fisher Scientific: 7-AAD viability staining solution (#00-6993-50), APC-conjugated anti-mouse CD11b (#17-0112-83), PE-conjugated anti-mouse F4/80 (#12-4801-80), FITC-conjugated anti-mouse CD40 (#11-0402-81), FITC-conjugated anti-mouse CD86 (#11-0862-81) and anti-mouse CD16/CD32 (#MFCR00). EDTA (#03690) was purchased from Sigma‒Aldrich.

### Animals

Female C57BL/6 mice (6‒8 weeks old) were purchased from the Hunan Laboratory Animal Center. All mice were housed in specific pathogen-free animal facilities at Jiangxi University of Traditional Chinese Medicine, and the mice experiments used protocols that were approved by the Institutional Animal Care and Use Committee.

### LPS-induced sepsis

We randomly divided mice into different groups, pretreated them for 2 h with either carvacrol or 0.2% Tween 80, and finally administered LPS by intraperitoneal injection. Mice were intraperitoneally injected with 10 mg/kg body weight LPS, peritoneal cells and serum were collected after 6 h and 24 h, and the liver and lung were collected after 12 h for haematoxylin and eosin staining. The survival after administration of LPS (25 mg/kg of body weight) was also monitored.

### Bone marrow-derived macrophage preparation

Bone marrow (BM) cells were isolated from the tibia and femur of C57BL/6 mice and cultured in complete Dulbecco's Modified Eagle Medium (DMEM) supplemented with M-CSF (10 ng/mL). Half of the medium was replaced with fresh DMEM supplemented with M-CSF (10 ng/mL) every other day. On day 5 of the culture, bone marrow-derived macrophages (BMDMs) were harvested and seeded at a density of 1 × 10^6^ cells/mL in fresh complete DMEM for experiments. These BMDMs were treated with carvacrol (5 μg/ml) for 2 h and then stimulated with LPS (1 μg/mL) at different times. The mRNA expression of proinflammatory cytokines was detected at 6 h; the protein expression of proinflammatory cytokines was detected at 24 h; and the phosphorylation of p38, ERK1/2, JNK, and p65 was detected at 10 min, 15 min and 24 h after LPS stimulation.

### Preparation of splenic macrophages

Spleens were placed into a plastic culture dish containing DMEM and were cut into several sections with scissors. The spleen was placed on a 70 µm filter screen and ground with the plunger of a 5 ml syringe. To lyse red blood cells, splenic cells were collected and incubated with 3 ml of 1 × lysing buffer for 2 min and washed with washing buffer (PBS containing 2% FBS and 5 mM EDTA) twice. Then, the cells were stained with 7-AAD viability staining solution for 10 min, followed by a mixture of anti-mouse CD16/CD32, APC-conjugated anti-mouse CD11b, and PE-conjugated anti-mouse F4/80 for 30 min on ice.

### Echocardiography

After LPS administration for 12 h, mice were anaesthetized with 2% isoflurane. Echocardiography was performed using ultrahigh-resolution small animal ultrasound (Visual Sonics, Canada, VEVO2100). Parameters of cardiac function were measured on the M-mode images, and the ejection fraction (EF) and fractional shortening (FS) data were collected.

### Real-time quantitative PCR (RT-qPCR)

We extracted RNA using TRIzol reagent (Invitrogen) according to the product instructions and performed qRT‒PCR using SYBR Green PCR Master Mix (Vazyme Biotech, China). The specific primer sequences used were as follows: 5′-TTCCATCCAGTTGCCTTCTTG-3′ and 5′-GAAGGCCGTGGTTGTCACC-3′ for mouse IL-6, 5′-AAGCCTGTAGCCCACGTCGTA-3′ and 5′-GGCACCACTAGTTGGTTGTCTTTG-3′ for TNF-α, and 5′-TGCTCGAGATGTCATGAAGGAG-3′ and 5′-CAGAGGGCCACAATGTGATG-3′ for mouse HPRT. The levels of the targeted genes were normalized to the level of HPRT.

### Cytokine assays

For cytokine assessment, we used mouse ELISA kits (Thermo-Fisher, USA) to measure the concentrations of IL-6 and TNF-α in the mouse serum, peritoneal lavage fluid, and culture supernatants according to the manufacturer’s recommended protocol.

### Western blot analysis

Cells were harvested and lysed, protein concentration was quantified by a bicinchoninic acid (BCA) protein assay kit, and the proteins were loaded on 10% SDS‒PAGE gels with equal amounts of protein per well. Subsequently, the proteins were transferred to PVDF membranes, which were then blocked with 3% bovine serum albumin (BSA) for 2 h at room temperature on a rotary shaker. The specific primary antibody was diluted in Tris-buffered saline with 0.1% Tween 20 (TBST) before being used for incubation with the membrane overnight at 4 °C, and then the membrane was incubated with the secondary antibody for 1 h at room temperature. Finally, detection was performed by using an enhanced chemiluminescence (ECL) western blot detection kit.

### Histological analysis

After 12 h of LPS treatment, mouse lung , liver and heart samples were collected and fixed with 4% paraformaldehyde overnight and embedded in paraffin, and the embedded lung, liver and heart tissues were sliced (5 μm) and then stained. The degree of lung injury was scored from 0 to 3 among the following domains^36^: haemorrhage, lung oedema, inflammatory cell infiltration, hyaline membrane, and atelectasis (0: “absent,” 1: “mild,” 2: “moderate,” and 3: “severe”). The degree of liver injury was scored from 0 to 3 based on the following parameters^37^: cytoplasmic colour fading, vacuolization, nuclear condensation, nuclear fragmentation, nuclear fading, and erythrocyte stasis (0: “absent,” 1: “mild,” 2: “moderate,” and 3: “severe”). The degree of heart injury was scored from 0 to 4 in sections containing the right and left ventricles, as follows: 0: “no injury or no inflammatory infiltrates”, 1: “isolated myocyte injury”, 2 : “one focal area of injury”, 3 : “two or more areas of injury” and 4 : “diffuse areas of damage compromising more than 50% of the myocardium”. The total injury score was then calculated by adding the scores for all parameters. Blind analysis was performed for all samples.

### Statistical analysis

Statistical analysis was performed using GraphPad Prism 6.0. All values are given as the means plus or minus standard error of the mean (SEM). Results with P < 0.05 were considered significant. We used one-way analysis of variance (ANOVA) and Tukey’s multiple comparisons test to compare the differences in one factor among three or more independent groups. When there were multiple factors, we used two-way ANOVA with a Bonferroni post-test.

### Ethics statement

The animal study was approved by the ethics committee of Jiangxi University of Traditional Chinese Medicine (No. JZLLSC20220794). All methods were carried out in accordance with relevant guidelines and regulations. All methods are reported in accordance with ARRIVE guidelines.

### Supplementary Information

Supplementary Information 1.

Supplementary Figures.

## Data Availability

All data generated or analysed during this study are included in this published article (and its Supplementary Information files S1).
