# Supplementary material for: Carvacrol protects mice against LPS-induced sepsis and attenuates inflammatory response in macrophages by modulating the ERK1/2 pathway
Source: Sci Rep. 2023 Aug 7;13:12809. doi: 10.1038/s41598-023-39665-7 (PMC10406886; doi:10.1038/s41598-023-39665-7)
Supplement: Supplementary file 1 — Supplementary Information 1. [file 41598_2023_39665_MOESM1_ESM.pdf]

1. The appropriate ethics declarations were added, please see “Ethics statement” (The animal study was approved by the ethics committee of Jiangxi University of Traditional Chinese Medicine (No. JZLLSC20220794). All methods were carried out in accordance with relevant guidelines and regulations. All methods are reported in accordance with ARRIVE guidelines.)

2. Full-length gels and blots are list below and the grouping of gels/blots cropped from different parts of the same gel, or from different gels have been described in the figure legend of Figure 5.

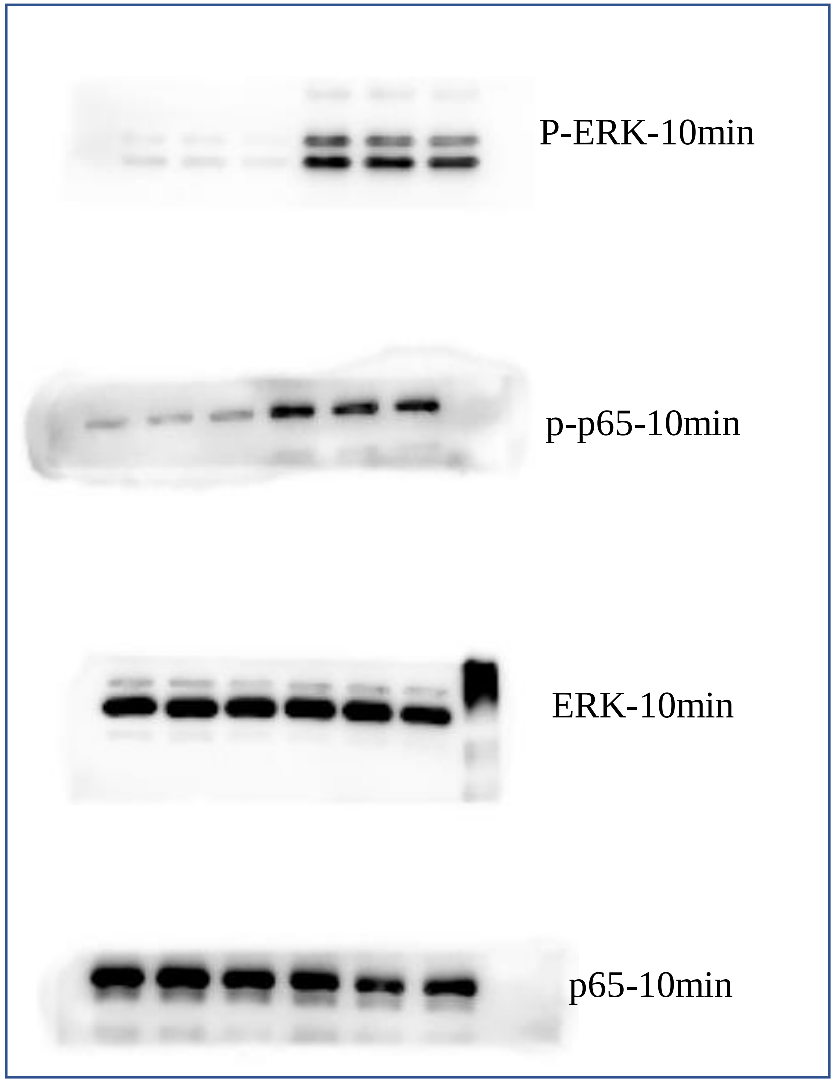

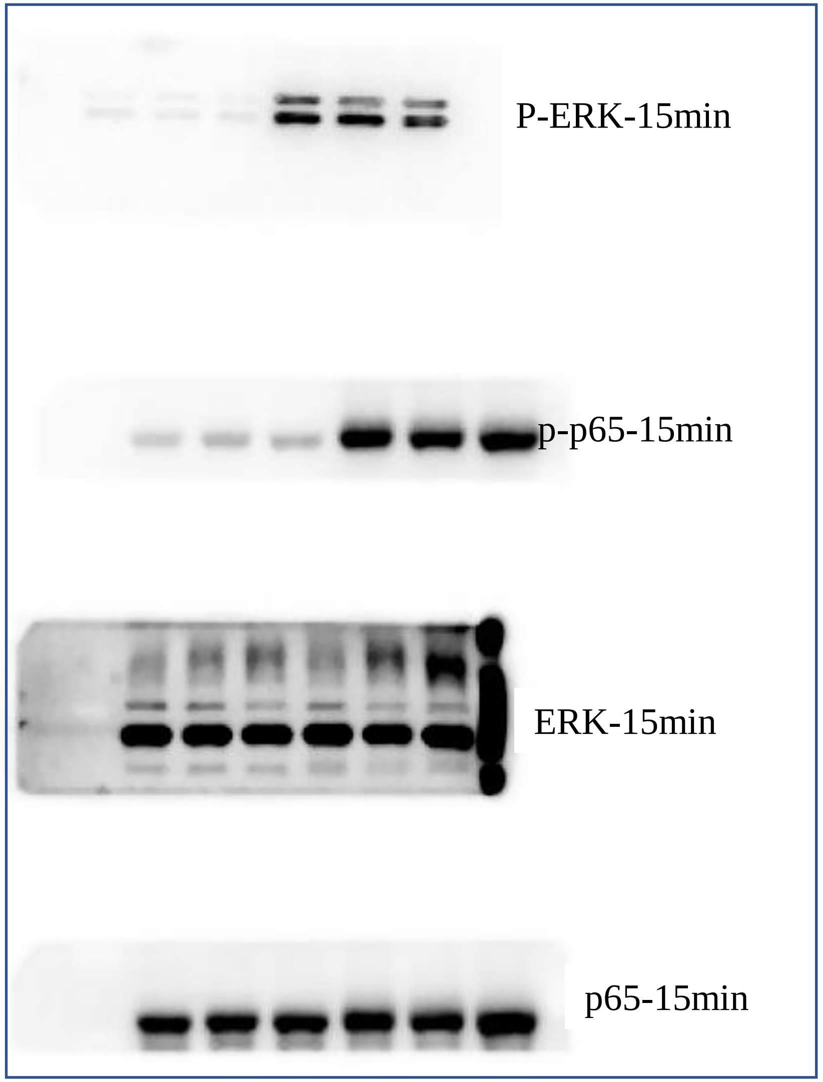

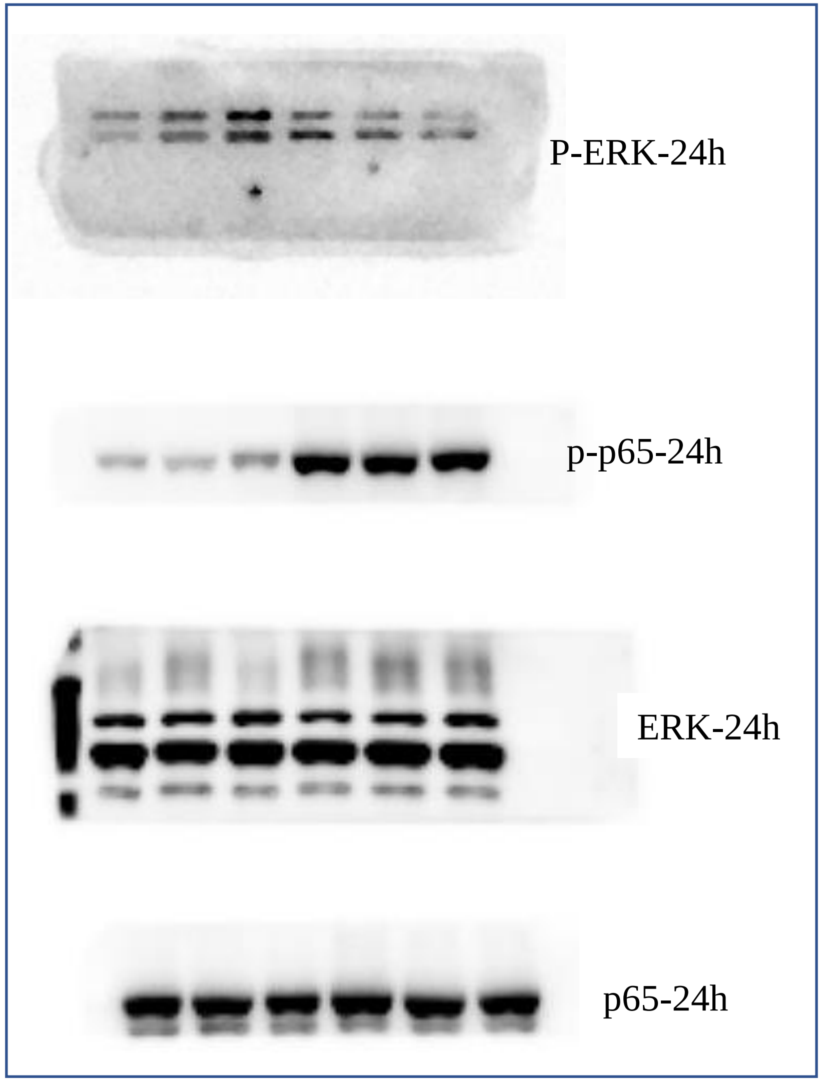

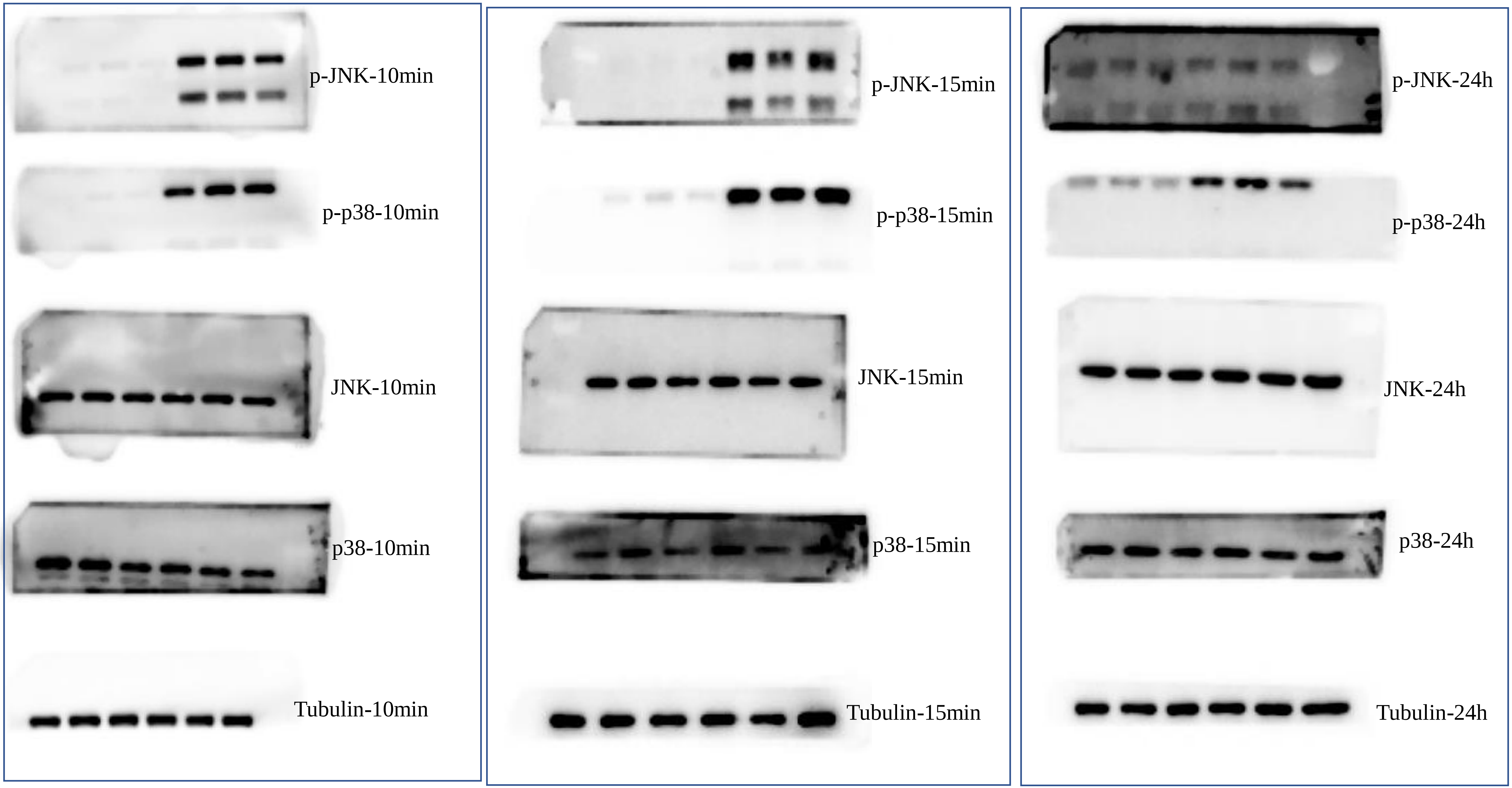
p-JNK-10min
p-p38-10min
JNK-10min
p38-10min
Tubulin-10min
p-JNK-15min
p-p38-15min
JNK-15min
p38-15min
Tubulin-15min
p-JNK-24h
p-p38-24h
JNK-24h
p38-24h
Tubulin-24h
