# Supplementary material for: Carvacrol protects mice against LPS-induced sepsis and attenuates inflammatory response in macrophages by modulating the ERK1/2 pathway
Source: Sci Rep. 2023 Aug 7;13:12809. doi: 10.1038/s41598-023-39665-7 (PMC10406886; doi:10.1038/s41598-023-39665-7)
Supplement: Supplementary file 2 — Supplementary Figures. [file 41598_2023_39665_MOESM2_ESM.docx]

Fig. S1 Treatments applied in the experiment. CAR: carvacrol, LPS: lipopolysaccharide.


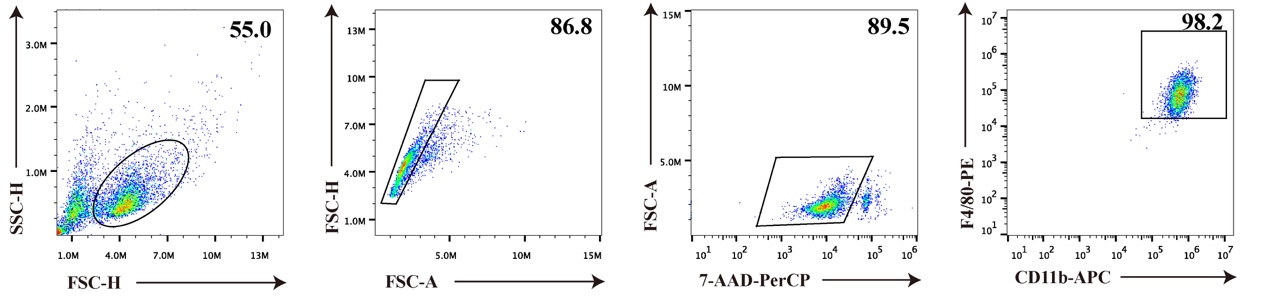


Fig. S2 Gating strategy for BMDMs that differentiated and were cultured for 6 days in vitro. BMDMs: CD11b^+^F4/80^+^ cells gated from live cells.
